# Advances in Developing Therapies to Combat Zika Virus: Current Knowledge and Future Perspectives

**DOI:** 10.3389/fmicb.2017.01469

**Published:** 2017-08-03

**Authors:** Ashok Munjal, Rekha Khandia, Kuldeep Dhama, Swati Sachan, Kumaragurubaran Karthik, Ruchi Tiwari, Yashpal S. Malik, Deepak Kumar, Raj K. Singh, Hafiz M. N. Iqbal, Sunil K. Joshi

**Affiliations:** ^1^Department of Biochemistry and Genetics, Barkatullah University Bhopal, India; ^2^Division of Pathology, ICAR-Indian Veterinary Research Institute Bareilly, India; ^3^Immunology Section, ICAR-Indian Veterinary Research Institute Bareilly, India; ^4^Central University Laboratory, Tamil Nadu Veterinary and Animal Sciences University Chennai, India; ^5^Department of Veterinary Microbiology and Immunology, College of Veterinary Sciences, UP Pandit Deen Dayal Upadhayay Pashu Chikitsa Vigyan Vishwavidyalay Evum Go-Anusandhan Sansthan Mathura, India; ^6^Division of Biological Standardization, ICAR-Indian Veterinary Research Institute Bareilly, India; ^7^Division of Veterinary Biotechnology, ICAR-Indian Veterinary Research Institute Bareilly, India; ^8^ICAR-Indian Veterinary Research Institute Bareilly, India; ^9^School of Engineering and Science, Tecnologico de Monterrey, Campus Monterrey Monterrey, Mexico; ^10^Cellular Immunology Lab, Frank Reidy Research Center of Bioelectrics, Old Dominion University, Norfolk VA, United States

**Keywords:** Zika virus, drugs, therapies, microcephaly, Guillain-Barré Syndrome

## Abstract

Zika virus (ZIKV) remained largely quiescent for nearly six decades after its first appearance in 1947. ZIKV reappeared after 2007, resulting in a declaration of an international “public health emergency” in 2016 by the World Health Organization (WHO). Until this time, ZIKV was considered to induce only mild illness, but it has now been established as the cause of severe clinical manifestations, including fetal anomalies, neurological problems, and autoimmune disorders. Infection during pregnancy can cause congenital brain abnormalities, including microcephaly and neurological degeneration, and in other cases, Guillain-Barré syndrome, making infections with ZIKV a substantial public health concern. Genomic and molecular investigations are underway to investigate ZIKV pathology and its recent enhanced pathogenicity, as well as to design safe and potent vaccines, drugs, and therapeutics. This review describes progress in the design and development of various anti-ZIKV therapeutics, including drugs targeting virus entry into cells and the helicase protein, nucleosides, inhibitors of NS3 protein, small molecules, methyltransferase inhibitors, interferons, repurposed drugs, drugs designed with the aid of computers, neutralizing antibodies, convalescent serum, antibodies that limit antibody-dependent enhancement, and herbal medicines. Additionally, covalent inhibitors of viral protein expression and anti-Toll-like receptor molecules are discussed. To counter ZIKV-associated disease, we need to make rapid progress in developing novel therapies that work effectually to inhibit ZIKV.

## Introduction

Zika virus (ZIKV) is a mosquito-borne virus belonging to the Spondweni serocomplex in the genus *Flavivirus* of the family *Flaviviridae* that has become a new threat following the Ebola virus epidemic (Singh et al., 2016). The expanding ZIKV epidemic was declared an emergency by the World Health Organization on February 1, 2016 (Fajardo et al., 2016; WHO, 2016). ZIKV is a single-stranded RNA virus that encodes a single polyprotein that is cleaved to form mature proteins, i.e., the capsid, envelope (E), and precursor of membrane and non-structural proteins. Other flaviviruses such as dengue virus (DENV), yellow fever virus (YFV), and West Nile virus (WNV) are closely related to ZIKV. In the last six decades since its discovery, ZIKV has been considered a mild human pathogen, but recently it has emerged as threat to global health, showing increased virulence, rapid spread, and an association with microcephaly and grave neurological complications like Guillain-Barré syndrome (GBS) (Cao-Lormeau et al., 2016; Carteaux et al., 2016; Mlakar et al., 2016; Sarno et al., 2016).

Zika virus has a wide tissue tropism in an experimental rhesus macaque model, infecting the hemolymphatic system, lymph nodes, spleen, cardiopulmonary, gastrointestinal, integument, and genitourinary tissues, along with the adrenal gland, spinal cord, and cerebrospinal fluid (Coffey et al., 2017). Additionally, it has been reported in muscles, kidneys, bladders, and in excreted urine (Gourinat et al., 2015). In males, ZIKV can infect testes (Govero et al., 2016), prostate and seminal vesicles, explaining the long-term persistence of viremia in semen, even after virus is no longer detectable in blood. In the female reproductive system, virus can be maintained in the vagina, uterus (Hirsch et al., 2017), vaginal epithelium (mice), and in uterine fibroblasts (Miner and Diamond, 2017). Miner and Diamond (2017) demonstrated the wide tissue tropism of the virus in Hofbauer cells, trophoblasts, and endothelial cells from the placenta. In addition, ZIKV was found to infect the cornea, neurosensory retina, optic nerve, aqueous humor, and tears. ZIKV infection in eyes results in uveitis (Furtado et al., 2016), and the persistence of the virus in cerebrospinal fluid and lymph nodes appears to enhance activity of rapamycin (mTOR), proinflammatory, and anti-apoptotic signaling pathways and reduce extracellular matrix signaling (Aid et al., 2017).

Zika virus adapts to human hosts by altering NS1 codon usage to facilitate viral replication and to increase viral titers (de Melo Freire et al., 2015). Furthermore, ZIKV placental transfer and its ability to infect neuronal tissue of growing fetuses is evident (Martines et al., 2016; Mlakar et al., 2016).

The complications of ZIKV infection are intensified by the unavailability of effective prophylactics, vaccines, or therapeutics. The spread of ZIKV, which, earlier, was limited to small geographical areas, has been facilitated by globalization, unplanned urbanization, poor sanitation, inadequate health services, and the emergence of insecticide resistance in mosquito vectors. Mosquitoes, mainly *Aedes aegypti* and *Ae. albopictus*, play a primary role in ZIKV transmission (Musso and Gubler, 2016). In addition, sexual transmission; male-to-female, female-to-male, and male-to-male transmissions have been reported by Hamer et al. (2017). A mathematical modeling study conducted by Gao et al. (2016) indicated that sexual activity contributed to 3.044% of transmission. During the typical incubation period of 2–7 days, despite the relatively low viral loads in people, infected human patients serve as a source of ZIKV (Foy et al., 2011). After viremia declines, convalescence begins, during which time a person is no longer infectious to a mosquito; however, they remain infective to other human hosts, with a low infection rate. The convalescent stage ends with establishment of long lasting immunity (Gao et al., 2016).

Zika virus vaccines in development include inactivated virus, nucleic acid-based vaccines (DNA or RNA), live vector vaccines, subunit vaccines, virus-like particles, and recombinant ZIKV. Because of its devastating effects, effective therapeutic agents and a vaccine are urgently needed. Presently, there are several drugs reported to be useful in treating ZIKV, a few of which are repurposed drugs. Efforts to develop effective drugs have increased worldwide, and a few compounds are in phase I trials (Alam et al., 2017; Ali et al., 2017). The present review discusses recent advances in and prospects for the design and development of various anti-viral drugs and therapeutics for ZIKV infection, including the identification of novel drug targets. The updated information compiled here will contribute to the design and development of additional effective drugs and pharmaceuticals to curtain the ill effects of ZIKV.

## Advances in the Design and Development of Anti-Zikv Drugs and Therapies

Specific anti-viral drugs are not yet available to combat ZIKV. Acetaminophen is used to control fever and pain, anti-histamines are used for pruritic rashes, and fluids are administered to prevent dehydration in ZIKV-infected patients. However, certain drugs such as acetylsalicylic acid (aspirin) and non-steroidal anti-inflammatory drugs (NSAIDs) are contraindicated because they increase the risk of internal bleeding, and other flaviviral infections, including DENV and chikungunya virus, can cause hemorrhage (; Mukherjee and Era, 2016; Musso and Gubler, 2016). ZIKV actively replicates in and causes death of neurons. Research for developing anti-viral drugs for Zika is going on fast and compounds like Sofosbuvir, 7-DMA, BCX4450, and NITD008 are currently entering a phase I trial (Ali et al., 2017).

Recently, several drugs and therapeutic candidates have been explored to determine the most effective treatment regimens to combat ZIKV and its severe clinical complications, which are being described in the following sections.

### Interferons as Anti-virals

Activation of the innate immune system by viruses leads to the release of interferons (IFNs), which are responsible for the elimination of viruses and for immune regulation. In an *in vitro* cell culture system developed for ZIKV cultivation, IFN-α, IFN-β, and IFN-γ have been shown to inhibit viral replication (Contreras and Arumugaswami, 2016). Type I interferons have shown dose-dependent inhibition of ZIKV replication in a cell culture study that used quantitative RT-PCR (Goebel et al., 2016). The inverse has been documented by Bowen et al. (2017); they demonstrated ZIKV’s ability to evade in the presence of type I interferon responses by degrading STAT2 signaling molecules. Trophoblastic cells secrete IFN-λ1, which exhibits anti-viral activities against single-stranded RNA viruses. In an *in vitro* model, conditioned medium obtained from PHT cells has been found to inhibit ZIKV growth in trophoblastic and non-trophoblastic cells by stimulating the secretion of IFN-λ1 (Bayer et al., 2016).

### Inhibition of Virus Entry into Cells

Inhibition of viral entry into a cell can serve as the first line of defense against ZIKV infection. ZIKV first binds to cell receptors, including AXL (Nowakowski et al., 2016), DC-SIGN, Tyro3, TIM, and TAM (Hamel et al., 2015), and then enters cells by clathrin-dependent endocytosis. ZIKV entry is severely hampered in human microglial cell line (CHME3) by silencing the clathrin heavy chain (a component essential for clathrin-coated vesicle formation) and dynamin-2 (a GTPase, required to pinch off endocytic vesicle from the plasma membrane). TIM receptors mediate viral entry after binding with viral phosphatidylserine and phosphatidylethanolamine (Jemielity et al., 2013). TIM1-mediated entry of DENV-2, WNV, and EBOV is inhibited by duramycin-biotin, which has less profound hemolytic effects and does not exhibit cellular cytotoxicity (Richard et al., 2015). The same TIM1 receptors are also involved in ZIKV entry; therefore, these drugs can be evaluated for the prevention of ZIKV entry. Peptide (GQASNGVFVIHWGKFDSFGIAV) derived from the Japanese encephalitis virus (JEV) E protein stem is able to prevent ZIKV infection with IC_50_ even at the nanomolar scale (3.93 nM). It also decreases the viral load and prevents histopathological damages in brain and testes in AG6 mouse, and attenuates the inflammatory response (Chen et al., 2017).

**FIGURE 1:**
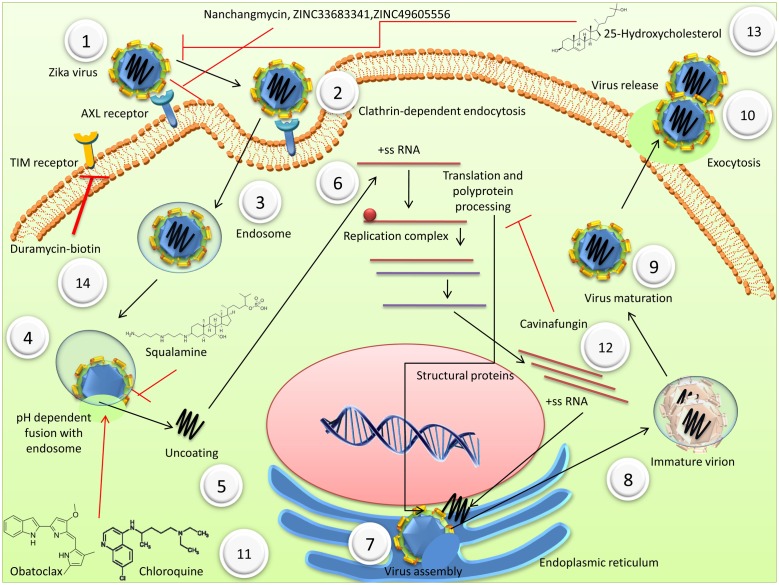
Mode of entry of Zika virus (ZIKV) and various drugs inhibiting viral entry and replication (1) ZIKV binds to cell receptors including AXL, DC-SIGN, Tyro3, TIM, and TAM. (2) Clathrin-dependent endocytosis. (3) Endosome mediated transport of ZIKV. (4) Fusion of virus membrane with host endosomal membrane, which depends on the pH. (5) Uncoating (6) The positive-sense genomic ssRNA is translated into a polyprotein, which is cleaved into all structural and non-structural proteins. Replication occurs at the surface of endoplasmic reticulum in cytoplasmic viral factories. A dsRNA genome is synthesized from the genomic ssRNA(+) (7) Virus assembly takes place at the endoplasmic reticulum. (8) At the endoplasmic reticulum, virions bud and are transported to the golgi apparatus. (9) In the golgi, prM protein is cleaved and maturation of the virion takes places. (10) Virions are released by exocytosis. (11) Obatoclax and chloroquineinhibit the acidic environment of endolysosomal vesicles. Squalamine, a cationic chemical, disturbs the electrostatic interaction between virus and host membranes during fusion and budding. (12) Cavinafungin, an alaninal-containing lipopeptide of fungal origin, inhibits ZIKV polyprotein processing and also the cleavage of signal peptide of host proteins. (13) Nanchangmycin, a polyether obtained from *Streptomyces nanchangensis*; small drug-like molecules, ZINC33683341 and ZINC49605556 block the receptor thus inhibiting the ZIKV entry. (14) TIM1 mediated entry is inhibited by Duramycin-biotin.

Modes of entry of ZIKV and various drugs inhibiting viral entry and replication have been depicted in **Figure 1**.

#### Blocking of Receptor Binding

After screening, more than 2000 molecules for their ability to inhibit ZIKV replication, nanchangmycin, a polyether obtained from *Streptomyces nanchangensis* that possesses insecticidal and anti-bacterial activity has been shown to block ZIKV replication in U2OS cells in *in vitro*. It is considered to act by targeting AXL receptors and blocking clathrin-mediated endocytosis. However, the exact mechanism of action of nanchangmycin is unknown (Rausch et al., 2017). Two small drug-like molecules, ZINC33683341 and ZINC49605556, both identified through homology modeling *in silico*, have been reported to inhibit ZIKV E protein by binding the viral receptors. Antiviral activities of ZINC33683341 have been confirmed in *in vitro* test. Thus, such viral inhibitors may be candidate molecules for ZIKV drugs after further research and clinical validation (Fernando et al., 2016).

#### Inhibition of Endosomal Fusion

Fusion of the endosome to lysosome is a critical step in releasing virus from endosomes. Obatoclax is a potential anti-neoplastic and pro-apoptotic synthetic small molecule Bcl-2 inhibitor. Its mesylate salt is reported to reduce the acidity of endolysosomal vesicles in *in vitro* model. Bcl-2 antagonists are effective only against viruses that require a low pH for fusion and entry, such as ZIKV, WNV, YFV, and others. Despite this limitation, Obatoclax works as a broad-spectrum anti-viral agent (Varghese et al., 2016). However, in clinical phase I and II trials while treating hematological and myeloid malignancies, Obatoclax did not produce satisfactory results, possibly due to inadequate inhibition of Bcl-2 family proteins. Chloroquine, which is an anti-malarial drug, raises endolysosomal pH and inhibits ZIKV infection in human brain microvascular endothelial cells, human neural stem cells, and mouse neurospheres (Delvecchio et al., 2016). Similarly, SaliPhe, a molecule under pre-clinical study and vATPase inhibitor, was tested as an inhibitor of endocytosis to obstruct ZIKV infection (Adcock et al., 2017). Griffithsin, a lectin isolated from the red alga *Griffithsia* sp., is a potent flaviviral entry inhibitor. It can cross-link high-mannose oligosaccharides present on the viral E glycoproteins and has shown wide anti-viral activity against HIV (Alexandre et al., 2011), HPV (Levendosky et al., 2015), HSV (Nixon et al., 2013), HCV (Takebe et al., 2013), and SARS (O’Keefe et al., 2010). Squalamine, a FDA approved cationic chemical, which act by disturbing electrostatic interactions between the virus and host membranes during fusion and budding (Zasloff et al., 2011), has been found well tolerated as component of eye drop in clinical studies conducted on human participants. Therefore, such potent drugs can be used as an anti-viral agent against ZIKV too.

### Inhibition of Virus Replication

The single-stranded RNA genome encodes a polyprotein, which is proteolytically cleaved into three structural proteins (C, prM, and E) and seven non-structural proteins (NS1, NS2A, NS2B, NS3, NS4A, NS4B, and NS5). The NS5 protein, an RNA-dependent RNA polymerase, plays an important role in viral RNA synthesis and inhibits IFN signaling by binding to STAT2 (Grant et al., 2016). ZIKV NS3 protein exhibits helicase activity that is essential for viral replication. The helicase domain of NS3 is activated by GTPγS (triphosphate), which facilitates the unwinding and translocation of RNA at the time of replication. The ZIKV helicase, along with NS5, is an attractive target for ZIKV drug development. Small membrane-associated interferon-inducible transmembrane proteins (IFITMs) are intrinsic immune system defenses that are able to inhibit replication of several pathogenic viruses. Both IFITM1 and IFITM3 have been reported to inhibit early stages of infection and replication of ZIKV in HeLa cells with the predominant role played by IFITM3 (Savidis et al., 2016). Cavinafungin, an alaninal-containing lipopeptide of fungal origin, has recently been found to inhibit ZIKV polyprotein processing and cleavage of host protein signal peptides through inhibition of host endoplasmic reticulum signal peptidase in *in vitro* model (Estoppey et al., 2017). Synthetic 25-hydroxycholesterol has been shown to inhibit ZIKV entry into the host in an *in vivo* assay using mouse and rhesus macaque models (Li et al., 2017). An *in vitro* study conducted in Vero cells using compounds such as ribavirin, CMX001, T-705, and T-1105 showed that T-705 (favipiravir) and T-1105 were able to reduce cell death caused by ZIKV (Cai et al., 2017). Thus, these compounds that inhibit ZIKV replication in cell culture need to be explored further so that they can be used safely against ZIKV.

**FIGURE 2:**
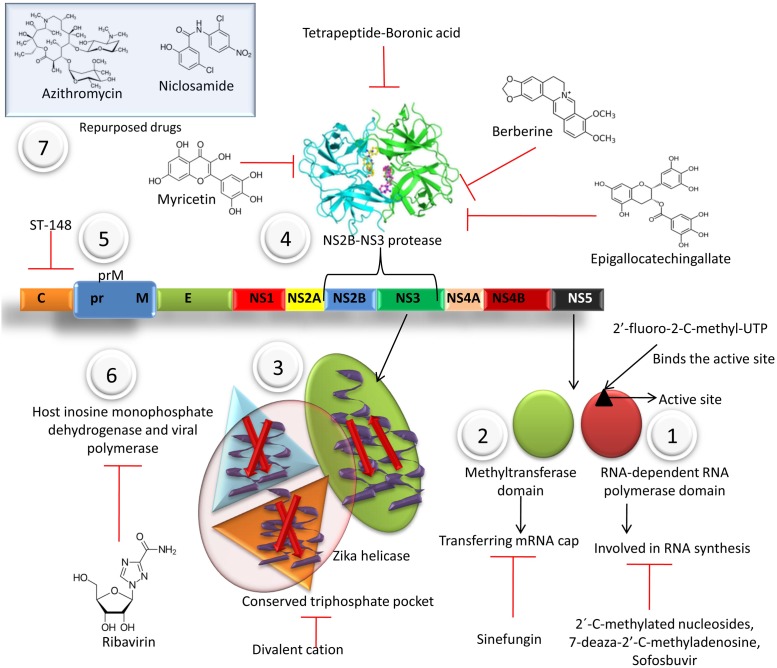
Various drugs involved in inhibition of virus replication at different stages. (1) Flaviviral NS5 has two major catalytic domains: RNA-dependent RNA polymerase (RdRp) and methyltransferase domain. Nucleoside analogs like 2′-C-methylated nucleosides, 7-deaza-2′-C-methyladenosine, Sofosbuvir may incorporate during the polymerase activity of RdRp in the viral nascent RNA chain and cause premature termination of RNA synthesis. The 2′-fluoro-2-C-methyl-UTP binds to the active site on NS5. (2) Methyltransferase domain is responsible for transferring mRNA cap. Sinefungin, an adenosine derivative, isolated from *Streptomyces griseoleus*, inhibit *S*-adenosyl-1-methionine (SAM), the natural substrate for methyltransferases and inhibit the methyltransferase activity. (3) Helicase crystal structure reveals a conserved triphosphate pocket and a positively charged tunnel for the accommodation of RNA. The helicase-activation is inhibited in the presence of divalent cation, due to extended conformation adopted by GTPγS in such conditions. (4) Tetrapeptide-Boronic acid is a potent inhibitor of NS2B-NS3 protease. Berberine, Myricetin, Epigallocatechingallate binds with affinity to NS3 protease and also inhibit the ZIKV replication. (5) Small-molecule inhibitor ST-148 inhibits capsid. (6) Ribavirin inhibits host inosine monophosphate dehydrogenase and viral polymerase. (7) Repurposed drugs like Chloroquine, azithromycin, niclosomide are used to treat ZIKV infection.

Numerous drugs involved in inhibition of virus replicationhave been portrayed in **Figure 2**.

#### Inhibition of NS5

The flaviviral NS5 protein has two major catalytic domains. The first domain is the RNA-dependent RNA polymerase (RdRp), and the second one is a methyltransferase domain (Egloff et al., 2002). Conserved regions in ZIKV NS5 MTase and RdRp structures have been identified, so that current anti-virals targeting these regions in flaviviruses like DENV, WNV, and YFV may be used against ZIKV (Duan et al., 2017). NS5 is also involved in IFN antagonism. Mutant ZIKV, which is unable to prevent IFN-stimulated signaling can grow in IFN-deficient cell lines and has vaccine potential. Structural analysis of NS5 will provide information to design small molecule inhibitors that may prevent NS5-mediated interferon inhibition (Dar et al., 2017).

##### Inhibition of the RdRp domain

Nucleoside analogs may be incorporated into the viral nascent RNA chain during RdRp activity, causing premature termination of RNA synthesis (Kok, 2016). After screening a library of nucleoside analogs, 2′-C-methylated nucleosides were found to be most efficient at terminating synthesis because they were selective for ZIKV, and there was no cytotoxicity or adverse effects on cellular proliferation observed (Eyer et al., 2016). These nucleosides do not target the active RdRp site; rather, they terminate elongation of the nascent viral RNA chain. Another nucleoside analog, 7-deaza-2′-C-methyladenosine, developed initially as an HCV replication inhibitor, was also evaluated for inhibition of ZIKV replication in Vero cells and a mouse model. Using a mouse model deficient in IFN-α/β and the IFN-γ receptor (AG129 strain), intraperitoneal inoculation of ZIKV with approximately 2 plaque-forming units (pfu)/animal resulted in disease. Inoculated animals were treated with 7-deaza-2′-C-methyladenosine at a dose of 50 mg/kg/day. The drug delayed the onset of viremia and virus-induced morbidity and mortality in infected mice (Zmurko et al., 2016). Despite demonstrating efficacy in animal models, the drug remained unsuccessful in the phase I clinical trial conducted. Adenosine analog NITD008 was found to be effective against flaviviruses including ZIKV in both *in vitro* and *in vivo* studies and exhibited reduced viremia in mice (Deng Y.Q. et al., 2016). Unfortunately, in pre-clinical animal testing, it was found to be too toxic to be suitable for human trials.

Sofosbuvir (Sovaldi) is a nucleotide analog inhibitor that is commercially available for the treatment of chronic HCV infection. Its active metabolite is 2′-fluoro-2-C-methyl-UTP, which binds to the active site of NS5 (Reznik and Ashby, 2017). It has also shown the ability to inhibit ZIKV infection in human tumor cell lines and human fetal-derived neuronal stem cells (Bullard-Feibelman et al., 2017; Reznik and Ashby, 2017). In clinical phase I and II studies, the regimen containing sofosbuvir was found to be clinically safe and efficacious (Mangia and Piazzolla, 2014). It is a class B drug and can be used in men and non-pregnant women to prevent tissue damage. The 2′-C-ethynyl and 2′-C-methyl analog of 5′-triphosphates were found to be incorporated by the RdRp of ZIKV and therefore to efficiently terminate the elongating RNA chain (Lu et al., 2016); hence, they may be candidate for the design of better anti-virals against ZIKV.

##### Methyltransferase domain inhibition

The NS5 protein contains methyltransferase, which is responsible for transferring the mRNA cap. An NS5 methyltransferase null mutant was found to be lethal for the virus. Targeting the NS5 methyltransferase structural domains may prevent ZIKV propagation (Zhang C. et al., 2016). Structural analysis of ZIKV-NS5 aided in the identification of methyltransferase inhibitors based on *in silico* analysis, which could lead to the identification of hotspots for drug designing and development of anti-ZIKV drugs (Coutard et al., 2016; Stephen et al., 2016). Furthermore, sinefungin, an adenosine derivative originally isolated from *Streptomyces griseoleus*, is a potential anti-fungal and anti-parasitic compound that competitively inhibits *S*-adenosyl-1-methionine (SAM), the natural substrate for methyltransferases, as well as methyltransferase activity. On the basis of structural computational analysis, sinefungin was able to attach to GTP and GDP analogs and might be useful in enhancing their affinity toward the enzyme for greater selectivity and inhibition of ZIKV replication (Hercik et al., 2017). Severe toxicity was observed with this drug in animal studies conducted in dogs and goats while its usage as an anti-parasitic agent (Robert-Gero et al., 1989), which has hampered its clinical use. Therefore, less toxic and better tolerated derivatives must be obtained and tested against ZIKV.

#### Inhibition of NS3 (Helicase)

An NS3 inhibitor can be used to impede ZIKV infection. The helicase action of NS3 is inhibited by divalent cations that cause GTPγS to adopt an extended conformation (Cao et al., 2016). An understanding of the interactions between NS3 and GTPγS has led to the identification of small molecules that inhibit ZIKV. The ZIKV helicase crystal structure has revealed a conserved triphosphate pocket and a positively charged tunnel that accommodates the RNA. This critical substrate-binding pocket may be a good target for anti-virals. Tian et al. (2016) reported that the helicase of ZIKV is closely related to those of other members of the family *Flaviviridae*. Therefore, drugs that target the helicase of these viruses could also be explored for the control of ZIKV.

#### Inhibition of the NS2B-NS3 Protease

The crystallographic structure of the NS2B-NS3 protease of ZIKV indicated that tetrapeptide-boronic acid would be a potent inhibitor of the NS2B-NS3 protease (Lei et al., 2016). Based on studies of surface plasmon resonance and the kinetics of protease inhibition, Lee et al. (2016) identified several small molecular inhibitors of the protease. In addition, the group identified a “pre-open conformation” for the ZIKV NS2B-NS3 protease by X-ray crystallographic analysis. A molecular docking analysis revealed that berberine, an FDA-approved quaternary ammonium salt used against DENV, has a high binding affinity to the NS3 protease of ZIKV (Sahoo et al., 2016). Further, a ‘Hybrid Combinatorial Substrate Library’ approach has also been used to determine the substrate specificity of the NS2B-NS3 protease for the design of phosphonate-containing protease inhibitors (Rut et al., 2016). Lim et al. (2017) tested 22 plant polyphenolic compounds for their ability to inhibit *Escherichia coli*-expressed ZIKV NS2B-NS3 pro-protease. Inhibition of protease activity was evaluated by a fluorescence resonance energy transfer-based assay. Among all compounds tested, myricetin showed the strongest inhibition of the NS2B-NS3 protease, followed by luteolin, epicatechin gallate, gallocatechin gallate, and epigallocatechin gallate. CN-716, a capped peptidomimetic boronic-acid compound, has been found to form ZIKV NS2B-NS3 protease inhibitor complex, which might have biological importance in inhibiting ZIKV replication (Lei et al., 2016). Structural and functional insights gained through crystallographic techniques will accelerate the discovery of structure-based anti-ZIKV compounds.

#### Nucleoside Biosynthesis Inhibitors

Nucleoside biosynthesis inhibitors have broad anti-viral activities, but limited numbers of such compounds are available for clinical use. Inhibition of host inosine monophosphate dehydrogenase (IMPDH) and viral polymerase is the key to the anti-viral activity of ribavirin against flaviviruses (Crance et al., 2003). Mycophenolic acid (MPA) is an IMPDH inhibitor and exhibited potent dose-dependent anti ZIKV activity (EC_50_ of <0.32 μM) in cell culture experiments that were confirmed using qRT-PCR (Goebel et al., 2016). Contrary to the above finding, Adcock et al. (2017) found MPA to be not very effective (EC_50_ >50 μM) with observation of significant cytotoxicity and cytopathogenic effects in a high-throughput assay. Dihydroorotate dehydrogenase (DHODH), a host enzyme which is responsible for pyrimidine biosynthesis, is a possible enzyme target for antiviral research. Brequinar, an inhibitor of this enzyme, exhibited anti-ZIKV activity with an EC_50_ (half maximal effective concentration, which is a common measure of a drug’s potency; the lower the EC_50_, the more effective the drug is) at submicromolar levels, but a low therapeutic index for Brequinar has restricted its clinical use.

#### Capsid inhibition

In comparison with targeting E protein, the capsid has gained less research attention. It is a dimeric protein with a positively charged surface and hydrophobic core pocket. A single small-molecule inhibitor (ST-148) has been identified, which, despite poor oral bioavailability, was shown to decrease the viremias and viral loads of DENV-1–4, Modoc virus, YFV, and HCV. ST-148 was screened in a panel of 20,000 chemically diverse molecules using a high-throughput assay. It is non-mutagenic and selectively inhibits flaviviruses (Byrd et al., 2013). ST-148 mediates the self-interaction of capsid proteins and imposes structural rigidity, disturbing the assembly and disassembly of DENV particles (Scaturro et al., 2014). The concept of capsid protein stabilization may also be applicable to ZIKV.

### Computer-Aided Drug Design

Before clinical studies, there are three essential phases of research: high-throughput computer or *in silico* drug design, medium-throughput *in vitro* drug testing, and low-throughput *in vivo* drug testing (Basak and Nandy, 2016). Understanding the ZIKV structure would aid in designing anti-viral therapies to curtail ZIKV infections (Cox et al., 2016). ZINC64717952 and ZINC39563464 have been found to block MTase and RdRp, respectively, based on a computational docking analysis (Ramharack and Soliman, 2017). The NS5 polymerase was inhibited by an andrographolide from *Andrographis*, whereas bisabolol and levomenol from *Matricaria recutita* and *Myoporum crassifolium*, respectively, blocked NS3 protease in virtual screening (Feranchuk et al., 2016). *In silico* techniques to identify enzyme blockers allow simultaneous assessments of various compounds with limits financial or experimental resource costs. A virtual screen of 36 million compounds from the MCULE database led to the selection of two molecules, MCULE-8830369631-0-1 and MCULE-9236850811-0-1, with inhibition constant (Ki) values of 0.08 and 0.30 μm, respectively (Onawole et al., 2017).

OpenZika, an IBM world community grid project, was used to identify drug molecule docking for various ZIKV structures. This platform allows data to be shared with researchers worldwide to facilitate the speedy discovery of anti-ZIKV drugs (Ekins et al., 2016). To elucidate therapeutically essential components like siRNAs, miRNAs, and sgRNAs (CRISPR/Cas9 targets) for ZIKV, an integrative multi-omics platform, ZikaVR^1^, is available. This platform offers other functions, including whole-genome alignment, codon information and bias assessments, phylogenetic deduction, and information regarding glycosylation sites and primer design (Gupta et al., 2016). The therapeutics based on enzyme, nucleoside, and capsid inhibitors are currently in their infancy and much more work needs to be carried out to bring these to clinical grounds. Computational analysis allows high throughput screening of potentially active molecules, however, *in vivo* validation is a prior requisite to move them from bench to bedside.

### Drug Repurposing

Drugs take decades to develop and test for efficacy and safety. Since there is presently no approved vaccine or drug available for ZIKV, the major focus of researchers, therefore, is on attempting drug repurposing. Scientists are evaluating repurposing of several FDA approved drugs against ZIKV infections. In this direction, a few promising drug candidates have been shortlisted by adapting various screening methodologies. For example, chloroquine, a 4-aminoquinoline, readily increases the pH of acidic vesicles (Akpovwa, 2016) and inhibits a conformational change essential for fusion between the virus envelope and endosomal membrane (Smit et al., 2011). *In vitro* studies revealed that chloroquine decreases the number of ZIKV-infected neural cells in different cell models and protects cellular death (Delvecchio et al., 2016). Other anti-malarial drugs such as quinacrine, mefloquine, and GSK369796 also demonstrate anti-ZIKV activity by inhibiting autophagy (Balasubramanian et al., 2016). During the screening of a library of FDA-approved drugs, both established anti-virals like bortezomib and mycophenolic acid and compounds with no previously reported anti-viral activity (e.g., daptomycin) were found to inhibit ZIKV replication in human cervical, placental, neural stem, and primary human amniotic cells (Barrows et al., 2016). Xu M. et al. (2016) screened a panel of compounds containing FDA-approved drugs, drugs in clinical trials, and pharmacologically active compounds to suppress infection-induced caspase activity. Human neural progenitor cells and glial SNB-19 cells infected with ZIKV were used as models to quantify ZIKV-induced caspase-3 activity. Of these compounds, a pro-caspase inhibitor, emricasan, successfully protected both neural cell monolayers and three-dimensional organoid cultures of neural cells by decreasing ZIKV-induced caspase-3. Similarly, screening of 725 FDA-approved chemically diverse compounds in ZIKV-infected Huh7 cells at a 20-μM concentration led to the selection of lovastatin, a drug used to reduce cholesterol; 5-fluorouracil used as a cancer treatment; 6-azauridine, a broad-spectrum antimetabolite; palonosetron, which is used to treat chemotherapy-induced nausea and vomiting; and kitasamycin, a macrolide antibiotic. The selection criteria included a selectivity index, maximum activity, and the EC_50_ of compounds (Pascoalino et al., 2016).

Niclosamide, clinically given to treat helminths inefction, can protect ZIKV-infected cells and inhibit virus replication (Xu M. et al., 2016). Bortezomib and sorafenib are anti-cancer drugs possessing anti-viral activity and have been well tolerated in phase I clinical trials (Cheng et al., 2016). Azithromycin, a commercially available antibiotic, was also found to inhibit ZIKV proliferation in cultured brain cells, suggesting a possibly drug to prevent GBS and microcephaly (Retallack et al., 2016). Recently, bromocriptine, a drug indicated for the treatment of pituitary tumors, Parkinson’s disease, and type 2 diabetes, was shown to inhibit ZIKV replication *in vitro*, possibly by occupying the active site of the ZIKV-NS2B-NS3 protein. A fluorescence-based enzymatic assay also revealed that bromocriptine inhibits the activity of the ZIKV-NS2B-NS3 protease, possibly by occupying the active site pocket. In addition, bromocriptine, along with type I interferon, exhibits synergistic anti-ZIKV activity (Chan et al., 2017). Suramin is an approved drug used to treat trypanosomal human sleeping sickness and is available for prophylactic and therapeutic uses in children. It inhibits the early steps of ZIKV binding/entry and decreases the number of infectious ZIKV progeny virions (Albulescu et al., 2017; Tan et al., 2017). The safe pediatric anti-protozoan and anti-viral drug nitazoxanide was found to affect post-attachment steps of ZIKV infection at or below a 10 μM dose (Cao et al., 2017). The anti-ZIKV activity of nitazoxanide is not less than that of niclosamide (Xu M. et al., 2016), but poor absorption of niclosamide might reduce its utility.

Hyperactivation of the *N*-methyl-D-aspartate receptor (NMDAR), mediated by enhanced glutamate release, may result in the accumulation of high levels of Ca^2+^ in neurons, and this may further lead to apoptosis or necrosis of neural cells. Neurodegeneration in ZIKV disease possibly occurs due to the excitotoxicity of glutamate. FDA approved NMDAR antagonistic drugs to treat Alzheimer’s disease (namely memantine, MK-801, agmatine, and ifenprodil) were found to prevent neuronal cell death caused by ZIKV under *in vitro* conditions without reducing viral titers (Costa et al., 2017). Memantine was found to bind non-competitively with NMDAR, while blocking only the pathologically active NMDAR and leaving its physiological activity unaffected (Sirohi and Kuhn, 2017). Memantine is also listed in pregnancy category B drugs by the FDA, hence it could be used safely to reduce neurological complications associated with ZIKV infection (Sirohi and Kuhn, 2017). Practical usage of repurposing earlier approved drugs could help in formulating fascinating approaches to counter ZIKV and its associated complications for which purpose more research work is needed before moving into clinical trials.

### Development of Pregnancy-Safe Drugs

The ability of ZIKV to infect fetuses and cause severe disease requires the development of drugs that function during pregnancy and that are safe for both the pregnant mother and fetus. The drugs must be able to cross the placental barrier to reach the fetus and to cross the blood-brain barrier to reach neural cells, the main targets of ZIKV. Khandia et al. (2017) summarized FDA-approved category B drugs (adequate animal study data shows no risk to fetuses, but controlled studies on pregnant women are unavailable) and category C drugs (animal studies revealed few teratogenic effects on fetuses, but control studies on pregnant women are unavailable; however, the potential benefits of using the drug may outweigh the risks). The list contains several drugs including the FDA category B drugs sofosbuvir (Sacramento et al., 2017), azithromycin (Retallack et al., 2016), niclosamide (Xu M. et al., 2016), palonosetron (Pascoalino et al., 2016), mefloquine (Balasubramanian et al., 2016), and daptomycin B (Barrows et al., 2016), category C drugs chloroquine (Delvecchio et al., 2016), amodiaquine, quinacrine hydrochloride (Balasubramanian et al., 2016), auranofin, clofazimine, deferasirox, methoxsalen, micafungin, sertraline-HCl, fingolimod, ivermectin, digoxin (Barrows et al., 2016), and seliciclib (Xu M. et al., 2016), which could be repurposed for treating ZIKV infection.

### Use of Convalescent Serum

Recently, neutralizing activity of human convalescent serum against ZIKV has been demonstrated in a standard plaque reduction neutralization test (Li et al., 2016). Further, a decreased number of ZIKV-infected brain cells in ICR albino fetal mice were observed after treating pregnant mice intraperitoneally with convalescent serum. Furthermore, ZIKV-mediated caspase activity was reduced, indicating the utility of convalescent serum in limiting ZIKV infection and cell death. Convalescent serum also reversed thinning of the cortical plate (CP) and ventricular zone (VZ)/subventricular zone (SVZ) observed in the brains of ZIKV-infected fetal mice. Therefore, the use of convalescent serum for the treatment of ZIKV-infected pregnant women and whether it can protect against brain abnormalities in fetuses should be assessed. A study by Wang S. et al. (2016) demonstrated the suppression of ZIKV infection in pregnant mice with a reduction in caspase-3-activated cells using convalescent serum with high amounts of neutralizing antibodies. Convalescent serum also inhibited progenitor cell death in infected fetal brain tissue, thereby preventing microcephaly. ZIKV-confirmed convalescent human serum was able to neutralize multiple strains of infectious ZIKV or ZIKV RVPs, indicating that ZIKV is circulating as a single serotype (Wang S. et al., 2016). Additionally, antibodies present in convalescent serum can cross the placental, as well as the blood-brain barrier, of fetuses; thus, it is a good candidate for the treatment of infected pregnant women.

The convalescent serum should be free from ZIKV and other blood-borne pathogens prior to transfer, therefore heat treatment at 58.0 ± 1.0°C for 590 ± 10 min or solvent/detergent (S/D) treatment is commonly employed. S/D treatment with 1% (wt/wt) tri-n-butyl phosphate (TBP) and 1% (wt/wt) octoxynol-9 at 30.0 ± 1.0°C and pH 6.9–7.4 for 60 min completely inactivated ZIKV (Kühnel et al., 2017). A photochemical, amotosalen, quickly intercalates into DNA and RNA strands and forms covalent adducts with pyrimidine, thereby inhibiting replication and transcription of the virus. Plasma samples treated with amotosalen and UVA-light are safe for use in patients, as they are free from viable ZIKV particles, even in the presence of a detectable amount of ZIKV viral RNA (Aubry et al., 2016).

### Use of Neutralizing Antibodies

A human monoclonal (mAb) antibody against DENV named C10 has been found to neutralize ZIKV E protein. Using an electron microscope, C10-ZIKV interactions were studied in extracellular (pH 8), early (pH 6.5), and late endosomal (pH 5.0) stages. At all of the tested pHs, C10 bound ZIKV E protein at different positions. At pH 8.0, it bound at the intradimer interface; at pH 6.5, it bound the virus surface; and at pH 5.0, it blocks raft structure. Of note, different structural rearrangements of the virus were blocked by C10 antibodies as depicted in visualization under electron microscope (Zhang S. et al., 2016), suggesting its broad applicability at different stage of infection. Out of the panel of human mAbs derived from patients previously infected with ZIKV, ZIKV-117 mAb was found to broadly neutralize the African and Asian-American lineages of ZIKV. The mAb recognized the unique quaternary epitope on the E protein dimer-dimer interface and effectively reduced ZIKV infection, maternal-to-fetal transfer, and tissue pathology and mortality (Sapparapu et al., 2016).

Three-dimensional cryo-electron microscopy showed that ZIKV-117 Fabs cross-link with monomers in surface E glycoprotein dimers and between neighboring dimers, thereby preventing the structural reorganization of E protein monomers and requiring the formation of fusogenic E protein trimers (Hasan et al., 2017). Neutralizing antibody 2A10G6, which targets the highly conserved fusion loop region of flavivirus E proteins, binds with high affinity and can neutralize ZIKV in a mouse model (Dai et al., 2016). Moreover, of 13 human mAbs from a single ZIKV patient, two mAbs (Z23 and Z3L1) potently bound and neutralized ZIKV, but it did not cross react with any DENV strains (Wang Q. et al., 2016).

### Strategies to Limit Antibody-Dependent Enhancement (ADE)

Studies regarding the phenomenon of antibody-dependent enhancement (ADE), in which viremia is increased in the presence of pre-existing cross-reactive, poorly neutralizing antibodies against a heterologous flavivirus strain, are controversial. In few cases, pre-existing cross-reactive antibodies have shown to increase ADE, while in some experiments such antibodies exhibited therapeutic potential. For instance, preincubation of human myeloid cells (U937), which are poorly permissive for ZIKV, with convalescent serum obtained from subjects who resolved DENV infections, resulted in increased ZIKV infectivity (Dejnirattisai et al., 2016). These results were confirmed by Charles and Christofferson (2016), who used a DENV serotype 2-derived mAb (4G2) to demonstrate ADE in ZIKV infections. A contradictory finding is reported by Pantoja et al. (2017) who reported reduction in viremia in DENV-exposed rhesus macaques, when compared with naïve animals. Antibodies to WNV have also shown cross-reactivity with ZIKV E protein, resulting in viremias that were at least 35-fold higher than those of the controls, and studies to determine the role of anti-WNV antibodies in enhancing ZIKV revealed a pattern like that observed with anti-DENV antibodies. WNV may enhance ZIKV *in vitro* as well as *in vivo*; however, the amplitude of enhancement is less than that of DENV (Bardina et al., 2017). Because 4G2 is widely used as an anti-flavivirus mAb, the possibility of using this mAb for other flaviviruses such as JEV and YFV or to limit WNV mediated ADE of ZIKV should be considered.

The two EDI/II cross-reactive mAbs developed against ZIKV (ZKA78) and DENV (DV82) were tested for their capacity for ADE of DENV and ZIKV infection in animal models. Further, in an AG129 mouse model, wild-type mAbs ZKA78 and DV82 (without the LALA mutation), when administered prior to DENV-2 infection, resulted in severe disease and death of mice on the 5th day post-infection, suggesting that the DENV infections were affected by the presence of pre-existing ZIKV antibodies (Stettler et al., 2016). However, antibodies against the envelope dimer epitope 1 (EDE1) region were shown to neutralize ZIKV, in addition to all four DENV serotypes, indicating their potential immune-therapeutic potential in ZIKV infections (Swanstrom et al., 2016). The mouse mAb 2A10G6 reported to bind the conserved 98DRXW101 motif of the FL loop is a broadly neutralizing antibody. It not only neutralizes DENV1-4, but also protected A129 mice against ZIKV infection (Dai et al., 2016). Hence, it seems that the antigenic epitopes against which the antibody is generated is the deciding factor in developing/not developing ADE. The epitopes resulting in poor neutralization lead to ADE, while the strongly neutralizing antibodies have therapeutic potential. Information regarding common epitopes may be useful in determining strategies to limit ADE (Xu X. et al., 2016).

**FIGURE 3:**
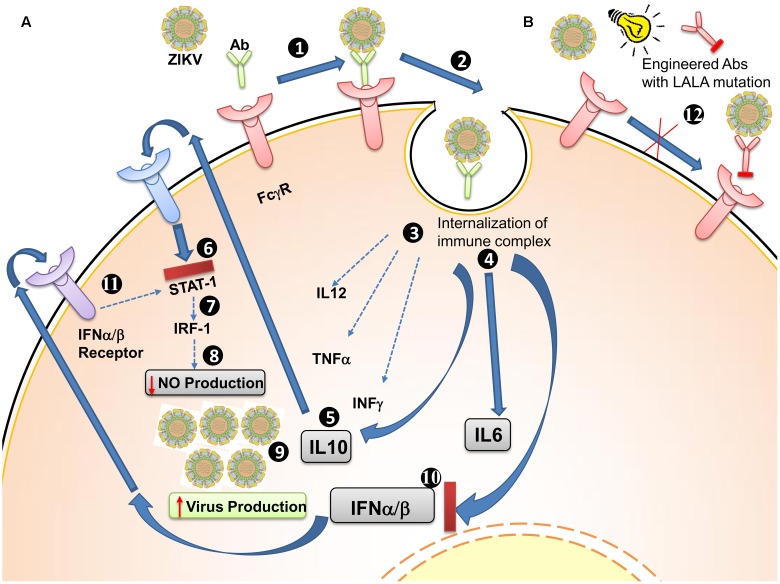
Antibody-dependent enhancement (ADE) of Zika virus (ZIKV) and strategies to limit it. **(A)** General ADE mechanism. (1) FcγRs are receptors present on the surface of various immune cells. Binding of antibody along with ZIKV results in (2) internalization of immune complexes into cells, (3) a reduction of IL12, TNFα, and IFNγ expression levels, and (4) increased levels of IL6 and IL12 (5). IL10 acts in an autocrine manner and binds to its own receptor (6), inhibiting the JAK-STAT pathway (7), which leads to reduced IRF1 production (8) and reduced IFN-stimulated response element (IRF1) production, resulting in decreased nitric oxide production. Nitric oxide, a diffusible radical antimicrobial and anti-viral, is reduced (9), which results in an increase in the number of infectious virus particles (10) IFNα/β are inhibited (11), diffuse out from the cell (12), and bind to their own receptors to reduce JAK-STAT signaling. **(B)** Antibodies engineered to prevent ADE (12) Engineered antibodies having LALA mutations in their Fc region are able to neutralize virus but fail to bind to FcγRs, thus preventing ADE.

To develop therapeutic mAb candidates, LALA mutations in the Fc region of antibodies have been examined (**Figure 3**). The LALA mutations are a leucine (L)-to-alanine (A) substitution at positions 234 and 235 (LALA) in the Fc region of IgG antibody. The binding of the Fc region of an antibody with gamma receptors (FcγRs), expressed on various immune cells, triggers their effector function. In the case of ZIKV or DENV, the virus-antibody immune complex is internalized by FcγRs and can result in ADE. Introduction of the LALA mutations into the Fc region abolishes its ability to bind to FcγRs (Arduin et al., 2015). Such engineered antibodies are incapable of interaction with FcγRs and hence eliminate ADE from prior DENV infections *in vitro* and *in vivo* (Williams et al., 2013).

Anti-ZIKV mAbs with the LALA mutation have been engineered for therapeutic and prophylactic purposes. The EDIII-specific neutralizing mAb ZKA64, which has the LALA mutations in the Fc region, blocked ADE in ZIKV infections in K652 cells in the presence of convalescent serum and completely protected A129 mice from lethal ZIKV challenge when administered one day prior to or post challenge (Stettler et al., 2016). Given the high potency and *in vitro* and *in vivo* efficacy of LALA mutant neutralizing antibodies, creation of such mutants appears to be a safe and promising approach to inhibit ADE in patients living in areas which co-circulating flaviviruses. The LALA mutations and substitution at amino acid position 297 (N297A) in the Fc region of an antibody reduces binding with FcγRs and C1q complement. Thus, such engineered antibodies may play an important role in therapeutics and prophylaxis (Arduin et al., 2015). DENV human mAb SIgN-3C, which strongly neutralizes ZIKV when the LALA mutations are introduced, did not induce ADE, showed a reduced viral load in fetal organs, and prevented virus-induced fetal growth retardation. This indicates the prophylactic potential of the antibody (Kam et al., 2017). In addition, mAbs such as Z23 and Z3L1 (mentioned in above section), which specifically neutralize ZIKV only and neither bind to nor neutralize any of the four DENV serotypes, are also of great importance in limiting ZIKV-associated ADE. In fact, a patent has been granted to Baehner et al. (2015), who modified the Fc region of human antibody, reducing its affinity for FcγRs by 1.15 to 100 folds and resulting in the inhibition of signaling cascades that lead to downstream immune response such as ADE in the case of DENV and ZIKV infection.

### Use of Herbal Drugs

Herbal drugs are of increasing interest because of the development of anti-microbial resistance in microbes and owing to their cost effectiveness. Curcumin, a common food additive, is able to reduce ZIKV infectivity by hindering the virus binding to host cell in a dose- and time-dependent manner, without having adverse effects on cellular viability (Mounce et al., 2017). Recently, a Chinese semi-synthetic formulation from *Andrographis paniculata* named xiyanping, in combination with other antiviral and symptomatic treatments was administered to treat Zika fever in a patient admitted in Ganxian People’s Hospital. The patient recovered within 7 days after starting treatment that included this medication (Deng Y. et al., 2016). Quercetin, a flavonoid present in fruits, vegetables, leaves, and grains, has been found to inhibit Zika NS2B-NS3pro enzymatic activity in a dose-dependent manner. Commercially available quercetin has been reported to inhibit the ZIKV protease with an IC_50_ of 26.0 ± 0.1 μM (Roy et al., 2016).

*In vitro* studies with a polyphenol, (^_^)-epigallocatechingallate (EGCG), found in green tea have shown inhibition of ZIKV (Carneiro et al., 2016). The structures of ZIKV NS2B-NS3 protease, NS3 helicase, NS5 methyltransferase, and NS5-RdRp were generated by homology modeling using the BLOSUM80 scoring matrix by Byler et al. (2016) and molecular docking with virtual library of phytochemicals was carried out. Out of 2263 plant-derived secondary metabolites tested, 43 compounds docked with at least one of the ZIKV enzymatic proteins. Some of these include balsacone B from *Populus balsamifera*, kanzonol V from *Glycyrrhiza glabra*, cinnamoylechinaxanthol from Echinacea, cimiphenol from *Actaea racemosa*, and rosemarinic acid from *Rosmarinus officinalis*. Such common medicinal plants may serve as a source of herbal anti-virals. Such studies encourage the findings of structure-based drug (Byler et al., 2016). Several bioactive components, including alkaloids, flavonoids, saponins, tannins, terpenoids, essential oils, and herbs, such as *Azidarachta indica* and *Tinospora cordifolia*, have shown anti-flaviviral activities against DENV, JEV, and YFV infections. Such antiviral agents can also be explored for their efficacy against ZIKV and be used as complementary alternative medicine (Parida et al., 2002; Kiat et al., 2006; Meneses et al., 2009; Tang et al., 2012; Roy et al., 2015; Ching et al., 2016; Gómez-Calderón et al., 2017).

Compounds used for the treatment of various flaviviral diseases that are readily adaptable to ZIKV are presented in **Table 1**.

**Table 1:** Patents of novel innovations useful for the treatment of various flaviviral diseases [readily adaptable for Zika virus (ZIKV) treatment].

S. No.	Patent title	Patent number	Date of publication/Application	Legal Status	Inventors’ Reference
1.	Carba-nucleoside analogs for antiviral treatment	WO2009132123A1	29.10.2009	Application	Cho et al., 2009
2.	Carba-nucleoside analogs for antiviral treatment	US8012942B2	06-09-2011	Granted	Butler et al., 2001
3.	Inhibitors of Flaviviridae viruses	WO2011088345A1	21.07.2011	Application	Canales et al., 2011
4.	Compositions and methods for treatment of viral diseases	WO2008033466A2	20.03.2008	Application	Johansen et al., 2008
5.	Antiviral agents for treatment of Flaviviridae infections	US20040266723A1	30.12.2004	Application	Otto et al., 2004
6.	Methods and compositions for treating Flaviviruses and Pestiviruses	US6812219B2	02.11.2004	Granted	LaColla and Sommadossi, 2004
7.	Compounds and pharmaceutical compositions for the treatment of viral infections	US7951789B2	31.05.2011	Granted	Sommadossi et al., 2011
8.	Antibody Fc variants	US 8969526 B2	03.03.2015	Granted	Baehner et al., 2015


### Other Strategies

Certain compounds such as QL-XII-47 and QL-XII-54 are quinolines (covalent inhibitors of DENV) that act by inhibiting viral E and NS5 protein expression without significantly affecting the host housekeeping protein GAPDH. Due to structural similarities between flaviviral E and NS5 proteins, similar compounds can be employed to combat ZIKV infection (de Wispelaere et al., 2016). Host caspases have been found to mediate the lethality of multiple pathogenic agents, based on the HapMap Project of B lymphoblastoid cells from a cohort of persons of African, European, and Asian ancestry. Bithionol (an FDA-approved drug) inhibits caspases and has been found to be effective in reducing the negative effects of ZIKV and bacterial and plant toxins. Thus, elucidation of such host proteins that further disease can be used to design drugs for ZIKV (Leonardi et al., 2016). Nordihydroguaiaretic acid (NDGA) alters the lipid metabolism of a host by intervening in the sterol regulatory element binding protein (SREBP) pathway. More recently, inhibitors of the SREBP pathway, including NDGA and its methylated derivative tetra-O-methyl nordihydroguaiaretic (M_4_N), PF-429242, and fatostatin, have also been found to reduce WNV and ZIKV replication. These drug candidates may serve as effective anti-viral agents against ZIKV (Merino-Ramos et al., 2017).

After ZIKV binds to nerve cells, Toll-like receptor (TLR)-3 is activated, leading to the dysregulation of genes participating in neurogenesis, axon guidance, and differentiation. TLR3 agonist poly (I:C) and thiophenecarboxamidopropionate compounds act as high-affinity competitive inhibitors of TLR3, and prevented a reduction in the size of ZIKV-treated neurospheres (Dang et al., 2016). Metadichol, a nanoemulsion of policosanols, binds to the Vit D receptor and stimulates the immune system. It can displace viruses bound to the Vit D receptor, thereby blocking viral entry into host cells. Metadichol has shown activity against ZIKV, EBOV, SARS coronavirus, JEV, WNV, and YFV. It is sold as a nutritional supplement in some Asian countries and is well tolerated; hence, it can be used as a safe and broad-spectrum anti-viral agent (Raghavan, 2016). Similarly, testing of other broad-spectrum anti-viral agents for anti-ZIKV activity will aid in rapid drug discovery to combat this virus.

An overview of recent advances in the design of drugs and therapies for ZIKV is presented in **Table 2**.

**Table 2:** Therapies available/possible for ZIKV treatment.

S. No.	Strategy to combat ZIKV	Assessed molecules	Modus Operandi	FDA pregnancy category	Reference
1.	Substrate binding pocket of helicase protein	Conserved triphosphate pocketPositively charged tunnel for the accommodation of RNA	Inhibit ZIKV replication	–	Tian et al., 2016
2.	Inhibitors of NS3 protein	Protease activity essential for its replication	Inhibit ZIKV replication	–	Sahoo et al., 2016
		Presence of divalent cation	Inactivation of helicase domain by extended conformation of GTPγS		Cao et al., 2016
3.	Inhibition of NS2B-NS3 protease	Phosphonate inhibitor	Inhibit ZIKV replication	–	Rut et al., 2016
4.	Inhibition of viral entry	Obatoclax	Inhibit acidic environment in endolysosomal vesicles	–	Varghese et al., 2016
		ZINC33683341 and ZINC49605556	Bind with viral receptor and inhibit entry	–	Fernando et al., 2016
5.	Nucleoside inhibitors	2′-C-methylated nucleosides	Cause premature termination of RNA synthesis		Kok, 2016
		7-deaza-2′-C-methyladenosine		–	Zmurko et al., 2016
		Sofosbuvir		B	Govero et al., 2016
		2′-C-ethynyl analog of 5′-triphosphates		–	Lu et al., 2016
		Active metabolite 2′-fluoro-2-C-methyl-UTP	Bind to active site present on NS5	–	Reznik and Ashby, 2017
		NITD008 adenosine analog	Cause premature termination of RNA synthesis	–	Deng Y.Q. et al., 2016
6.	Interferon	Type I interferons- IFN-α and IFN-β	Antiviral defense system inhibits ZIKV replication	–	Schneider et al., 2014
		Human trophoblast (PHT) cell culture conditioned media secreting type III IFN and IFN-λ1	Inhibit ZIKV replication	–	Bayer et al., 2016
		IFN-α, β, γ	Inhibit ZIKV replication	–	Contreras and Arumugaswami, 2016
7.	Neutralizing antibodies	mAbto E proteindimer–dimer interface	Reduce ZIKV infection, maternal to fetal transfer and tissue pathology	–	Sapparapu et al., 2016
		mAb 2A10G6 to FLE region	Protect from ZIKV infection *in vivo*	–	Dai et al., 2016
		mAb ZIKV-117 to E region	Broadly neutralize several ZIKV lineages.	–	
		mAb Z23 and Z3L1	Inhibit ZIKV replication	–	
		mAb C10- against E region	Inhibit ZIKV replication	–	Zhang S. et al., 2016
8.	Convalescent serum	Polyclonal neutralizing high titer serum	Reduction in caspase activity	–	Li et al., 2016
			Prevent thinning of the cortical plate (CP) and ventricular zone (VZ)/ subventricular zone (SVZ)	–	
			Prevention of neural progenitor cell death in infected fetal brain tissue and prevention of microcephaly	–	Wang Q. et al., 2016
9.	Drug repurposing	Niclosamide	Inhibit ZIKV replication	B	Xu M. et al., 2016
		PHA-690509	Cyclin-dependent kinase (CDK) inhibitor studied for use in treating cancer; Known to interfere with gene expression	D	Barreyro et al., 2015; Xu M. et al., 2016
		Emricasan	Protect brain cells of developing fetuses against viral damage by inhibiting apoptosis	–	Xu M. et al., 2016
		Seliciclib	Cyclin-dependent kinase (CDK) inhibitor	D	Xu M. et al., 2016
		Bortezomib	Replication inhibition in human cervical, placental, neural stem and primary human amniotic cells	D	Barrows et al., 2016
		Mycophenolic acid		D	
		Auranofin	unknown	C	
		Vermectin	Antiparasitic; inhibits viral protein functioning	C	Mastrangelo et al., 2012; Barrows et al., 2016
		Daptomycin	Cause bacterial membrane depolarization and a potassium ion efflux.	B	Silverman et al., 2003; Barrows et al., 2016
		Sertraline	Inhibit phospholipase A1 and phospholipase D	C	Rainey et al., 2010; Barrows et al., 2016
		Pyrimethamine	Dihydrofolate Reductase Inhibitor and block purine and pyrimidine synthesis	C	Barrows et al., 2016
		Cyclosporine A	Immunosuppression by selectively inhibiting cytokine-induced DNA binding of activator protein-1 and NF-κB.	C	Doller et al., 2007; Barrows et al., 2016
		Azathioprine	Inhibits purine synthesis	D	Lennard, 1992; Barrows et al., 2016
		Vinblastine	Anticancer; Microtubule inhibitor	D	Kouznetsova et al., 2014
		Vinorelbine/Navelbine	Anticancer Microtubule inhibitor	D	
		Vincristine	Anticancer Microtubule inhibitor	D	
		Nocodazole	Anticancer Microtubule inhibitor	–	
		Sunitinib	Anticancer Kinase inhibitor	D	
		Toremifene	Anticancer Estrogen receptor modulator	D	
		Daunomycin	AnticancerTopoisomerase Inhibitor	D	
		Clemastine	Antiallergic, hay fever, rhinitis histamine antagonist	B	
		Digoxin	Antiarrhythmic Na^+^-K^+^ pump inhibitor	C	
		Colchicine	Primary for gout, microtubule inhibitor	C	
		Propafenone	Antiarrhythmic sodium channel blocker	C	
		Dronedarone	Antiarrhythmic multichannel blocker	X (not for use in pregnancy)	
		Maprotiline	Antidepressant adrenergic uptake inhibitors and histamine antagonist	B	
		Thiothixene	Antipsychotic dopamine antagonist	–	
		Clomipramine	Antidepressant serotonin uptake inhibitors and histamine antagonist	C	
		Trifluoperazine	Antipsychotic, antiemetic dopamine antagonist	–	
		Benztropine	Anticholinergic, antihistamine histamine antagonist and cholinergic antagonist	B2	
		Azithromycin	Antimicrobial protein synthesis inhibitor	B	
		Clarithromycin	Antimicrobial protein synthesis inhibitor	C	
		Mebendazole	Antihelminthic microtubule inhibitor	C	
		Albendazole	Anthelmintic microtubule inhibitor	C	
		Azithromycin	Antibacterial	B	Retallack et al., 2016
		Quinacrine, Mefloquine, and GSK369796	Antimalarial drug inhibiting autophagy	C	Balasubramanian et al., 2016
		Sinefungin	Antifungal antibiotic	Not approved	Hercik et al., 2017
		Suramin	African trypanosomiasis	Not approved	Albulescu et al., 2017
		Nitazoxanide	Antiprotozoan drug	B	Cao et al., 2017
		Memantine	For treating Alzheimer’s Diseases	B	Costa et al., 2017
		Kitasamycin	Broad spectrum antimicrobial;	–	Saiz and Martín-Acebes, 2017
		Lovastatin	Inhibits cholesterol 247 biosynthesis.	X	
		Nordihydroguaiaretic acid	Anticancer drug	Not apporoved	
		PF-429242	Impairs the onset of HCV infection.	–	
		Fatostatin	Fat synthesis blocker	–	
		6-azauridine	Inhibits *de novo* pyrimidine synthesis	D	Adcock et al., 2017
		Finasteride	For treatment of benign prostatic hyperplasia	X	
		Mycophenolic acid	Immunosupressant	D	Goebel et al., 2016
10.	Herbal therapies	Preparation of *Alternanthera philoxeroides*, *Andrographispaniculata*, *Azidarachtaindica*, *Euphorbia hirta*, *Eupatorium perfoliatum*, *Tinosporacordifolia*, and *Psidiumguajava*	Broad spectrum antiviral activity	–	Parida et al., 2002; Jiang et al., 2005; Apostol et al., 2012; Sriwilaijaroen et al., 2012; Tang et al., 2012; Ching et al., 2016; Saxena et al., 2016
		Anti-inflammatory drugs + *Andrographispaniculata*	Inhibit ZIKV replication	–	Deng Y.Q. et al., 2016
		Andrographolide	Inhibition of NS5 polymerase pocket	–	Feranchuk et al., 2016
		Bisabololand levomenol from *Matricariarecutita* and *Myoporumcrassifolium*	Inhibition of NS3 protease	–	
		Epigallocatechin gallate (EGCG) found in green tea	Inhibitory effect (Inhibition through yet unknown mechanisms)	–	Carneiro et al., 2016
		Quercitin and myricetin (flavonoid)	Allosteric inhibition of NS2B-NS3 protease	–	Lim et al., 2016, 2017; Roy et al., 2016
		Balsacone B, kanzonol V, cinnamoylechinaxanthol, cimiphenol and rosemarinic acid	Inhibitory effect (Inhibition through yet unknown mechanisms)	–	Byler et al., 2016
11.	Other strategies	Methyltransferase inhibitors	Inhibition of NS5	–	Stephen et al., 2016
		Methyltransferase inhibitor- QL-XII-47 and QL-XII-54	Inhibition of viral protein expression	–	de Wispelaere et al., 2016
		Bithionol	Inhibition of caspases	C	Leonardi et al., 2016
		Amotosalen combined with UVA light	Viral inactivation	–	Aubry et al., 2016
		Metadichol	Binds to Vitamin D receptor and displays virus	–	Raghavan, 2016


A few recent therapies include cytokines, TLRs, siRNA, RNA interference, probiotics, immunomodulatory interventions, and nanodrug delivery. These approaches have gained momentum and are being examined for optimum benefit and safety in humans and their companion animals. Prospective aspects of these valuable therapies could be given a due focus for designing and developing effective drugs, medicines, therapeutics and immunomodulatory pharmaceuticals for the treatment of ZIKV infections. Molecular and genetic analyses for a more in-depth understanding of ZIKV pathogenesis would facilitate the identification of novel targets and development of safer and more effective drugs to counter ZIKV effectively.

## Conclusion and Future Perspectives

Zika virus, an arbovirus, shares several characteristic features with other members of the *Flavivirus* family Recent evidences of autoimmune complications (GBS) and maternal-to-fetal transmission of virus leading to microcephaly has accumulated. Using state-of-the-art methods to formulate effective diagnostics, anti-viral drugs, therapeutics, vaccines, and prevention and control strategies would aid in addressing this emergent virus. Some recent therapies have shown promise in inhibiting ZIKV infections and associated disease. These therapies include limiting viral entry into cells, targeting the ZIKV helicase protein, use of nucleoside analogs like 2′-C-methylated nucleosides and 7-deaza-2′-C-methyladenosine to terminate nascent RNA strand formation, and use of antibodies that bind to ZIKV but do not neutralize it, reducing the risk of ADE. ADE is of major concern in the application of ZIKV therapies in geographical regions where other flaviviruses are endemic. Thus, to limit ADE, antibodies are being engineering to contain a modified Fc region. Modification of the Fc region of antibodies not only hampers their attachment to FcγRs to inhibit internalization of the immune complex, but also reduces complement binding, preventing ADE. In the future, several such mutations may be identified, and humanized mAbs can be genetically engineered to prevent ADE. Combination use of such engineered antibodies might be evaluated for synergistic effects in other therapeutic and prophylactic regimens.

Encouraging results with repurposed drugs, as shown by the use of chloroquine, a malaria drug, has led to the screening of several other FDA-approved drugs, including niclosamide, emricasan, and daptomycin, palonosetron, kitasamycin, and many more, for ZIKV treatment. Another valuable strategy for the discovery of ZIKV preventives and anti-virals is the use of computational analysis.

More insights into genetic and molecular mechanisms associated with the recent increase in virulence of ZIKV could aid in the design and development of safer and more potent drugs and therapeutics against ZIKV. Along with identifying novel drug targets, therapeutics, and vaccines, strengthening of appropriate prevention and control measures, including mosquito control, could help in limiting ZIKV infections, its associated complications, and its potential for further spread. It is time for researchers, pharmaceutical companies, policy makers, regulators, and funding agencies to identify and implement strategies to counter ZIKV globally.

### What We Are Still Lacking

Since the declaration of the Zika epidemic as an international public health emergency by WHO in 2016, research on ZIKV has increased many fold. However, there are areas that still need to be addressed.

(1) The percent contribution of each route of ZIKV infection is not precisely understood. Presently, based on mathematical modeling study, sexual transmission has been estimated to account for upto 3% of transmission, but contributions by other routes of infection are yet to be studied. This knowledge may be helpful in designing precisely targeted inhibitory molecules to block infection at site of entry.

(2) Many FDA approved drugs have been tested for efficacy against ZIKV; which can be repurposed for treating ZIKV infection in human. However, to date, no FDA category A drug has been identified clinically safe for use in mothers and fetuses.

(3) For engineered mAb, only two mutations that prevent internalization of immune complexes, i.e., LALA and N297A substitutions, have been identified. More such mutations must be identified for optimal efficacy and synergism.

## Author Contributions

All the authors substantially contributed to the conception, design, analysis and interpretation of data, checking and approving final version of manuscript, and agree to be accountable for its contents. AM and RK initiated this review compilation; KD reviewed, analyzed, and edited; RK designed tables; AM, RK, and KK designed the figures; SS and RT covered critical aspects on drug and vaccine development; YM and RS reviewed virological aspects and analyzed data; DK reviewed biotechnological and bioinformatics advances; HI and SK overviewed immunotherapeutic aspects and drug development.

## Conflict of Interest Statement

The authors declare that the research was conducted in the absence of any commercial or financial relationships that could be construed as a potential conflict of interest.

## Footnotes

1 http://bioinfo.imtech.res.in/manojk/zikavr/
